# Genome Sequence of Paenibacillus polymyxa Strain HOB6, Isolated from Hemp Seed Oil

**DOI:** 10.1128/MRA.00344-21

**Published:** 2021-06-03

**Authors:** Mohannad Mahmoud, Suha Jabaji

**Affiliations:** a Department of Plant Science, McGill University, Montreal, Quebec, Canada; University of Arizona

## Abstract

Paenibacillus polymyxa strain HOB6 was isolated from hemp seed oil. The strain displays antimicrobial activity against fungal pathogens and has potential for development as a biopesticide against cannabis diseases. Its genome was sequenced and annotated, uncovering the ability to encode the biosynthetic pathways for antimicrobial lanthipeptides and nonribosomal peptides.

## ANNOUNCEMENT

*Paenibacillus* is a genus of facultative endospore-forming bacteria, and a majority of its strains are found in soil (1). *Paenibacillus* strains are known for their growth promotion attributes and the production of microbial arsenals relevant to agriculture and medicine (2, 3). Exploring the genome of Paenibacillus polymyxa HOB6 can provide a deep knowledge of the underlying mechanisms against a variety of phytopathogens, thus leading to lower usage of synthetic pesticides (4). Hemp seed oil was purchased from a commercial organic producer (Coco et Calendula, Inc., Montreal, Canada). An equal volume of seed oil was mixed with an equal volume of LB broth in a 15-ml Falcon tube and incubated with agitation (140 rpm) overnight at room temperature. An aliquot (100 μl) of the aqueous phase was streaked onto LB agar (LBA) medium and incubated at 37°C for 5 days to obtain pure single-cell colonies. Genomic DNA (gDNA) was extracted from a single-cell colony using a DNeasy blood and tissue kit (Qiagen, Germany). The preparation of the whole-genome shotgun library and sequencing were carried out by Admera Health (South Plainfield, NJ, USA). The gDNA was fragmented using a Covaris LE220 sonicator, and the paired-end sequencing library was prepared using a Nextera XT DNA library prep kit (Illumina, USA). Genome sequencing was performed on a HiSeq X platform, using a 2 × 150-bp protocol. A total of 5,243,904 sequencing reads were produced and uploaded to the Galaxy Web platform (5), and we used the public server at https://usegalaxy.org/ to analyze our data using default settings unless otherwise specified. Preprocessing of the sequencing reads was carried out using FastQC version 0.11.9 software (6) for quality assessment and Trim Galore (Galaxy version 0.6.3) (7) to remove the low-quality reads and adapters. The filtered reads were assembled *de novo* using SPAdes (Galaxy version 3.12.0) (8), with k-mer sizes of 21, 33, 55, 77, 99, 111, and 127, which resulted in a total of 219 contigs. Reference genome sequences belonging to five *P. polymyxa* strains were used to rearrange and correctly orient the assembly contigs into scaffolds using MeDuSa version 1.6 (9). These strains and their GenBank assembly accession numbers are as follows: CR1 (GCA_000507205.2), E681 (GCA_014706575.1), SC2 (GCA_000164985.2), SQR-21 (GCA_000597985.1), and HY96-2 (GCA_002893885.1). The resultant scaffolds were subjected to NCBI’s contamination screen, and contigs containing contaminants as well as those under 0.2 kb were removed. This resulted in a total of 59 scaffolds with a total length of 5,751,895 bp, a G+C content of 45.57%, and an *N*_50_ value of 2,906,550 bp, with an average depth of sequencing coverage of 266.29×. The assembly statistics were provided by Quast (Galaxy version 5.0.2) (10). Following the ribosomal multilocus sequence typing approach (https://pubmlst.org/species-id/) (11), strain HOB6 was identified as Paenibacillus polymyxa. The draft genome sequence was annotated using the NCBI Prokaryotic Genome Annotation Pipeline (PGAP) version 5.1 (12). Annotation of this genome revealed a total of 5,248 genes, including 4,956 coding DNA sequences (CDSs), 157 RNA genes, 107 tRNAs, 21 complete rRNAs, 4 noncoding RNAs (ncRNAs), and 135 pseudogenes. AntiSMASH version 5 (13) was used for biosynthetic gene cluster identification. The strain HOB6 genome comprises gene clusters for the nonribosomal peptides (NRPs) fusaricidin B, polymyxin, and tridecaptin, as well as the lanthipeptides paenicidin B and paenibacillin.

### Data availability.

This whole-genome shotgun project has been deposited at DDBJ/EMBL/GenBank under accession no. JAFJXZ000000000. The version described in this paper is the first version, JAFJXZ010000000. The sequencing reads have been deposited in the NCBI SRA under accession no. SRR13795614.

## References

[1] Daud NS, Mohd Din ARJ, Rosli MA, Azam ZM, Othman NZ, Sarmidi MR. 2019. *Paenibacillus polymyxa* bioactive compounds for agricultural and biotechnological applications. Biocatal Agric Biotechnol 18:101092. doi:10.1016/j.bcab.2019.101092.

[2] Grady EN, MacDonald J, Liu L, Richman A, Yuan Z-C. 2016. Current knowledge and perspectives of *Paenibacillus*: a review. Microb Cell Fact 15:203. doi:10.1186/s12934-016-0603-7. 27905924 PMC5134293

[3] Langendries S, Goormachtig S. 8 March 2021. Paenibacillus polymyxa, a jack of all trades. Environ Microbiol doi:10.1111/1462-2920.15450. 33684235

[4] Brühl CA, Zaller JG. 2019. Biodiversity decline as a consequence of an inappropriate environmental risk assessment of pesticides. Front Environ Sci 7:177. doi:10.3389/fenvs.2019.00177.

[5] Jalili V, Afgan E, Gu Q, Clements D, Blankenberg D, Goecks J, Taylor J, Nekrutenko A. 2020. The Galaxy platform for accessible, reproducible and collaborative biomedical analyses: 2020 update. Nucleic Acids Res 48:W395–W402. doi:10.1093/nar/gkaa434. 32479607 PMC7319590

[6] Andrews S. 2010. FastQC: a quality control tool for high throughput sequence data. Babraham Bioinformatics, Babraham Institute, Cambridge, United Kingdom. https://www.bioinformatics.babraham.ac.uk/projects/fastqc/.

[7] Krueger F. 2015. Trim Galore: a wrapper tool around Cutadapt and FastQC to consistently apply quality and adapter trimming to FastQ files. https://www.bioinformatics.babraham.ac.uk/projects/trim_galore/.

[8] Bankevich A, Nurk S, Antipov D, Gurevich AA, Dvorkin M, Kulikov AS, Lesin VM, Nikolenko SI, Pham S, Prjibelski AD, Pyshkin AV, Sirotkin AV, Vyahhi N, Tesler G, Alekseyev MA, Pevzner PA. 2012. SPAdes: a new genome assembly algorithm and its applications to single-cell sequencing. J Comput Biol 19:455–477. doi:10.1089/cmb.2012.0021. 22506599 PMC3342519

[9] Bosi E, Donati B, Galardini M, Brunetti S, Sagot M-F, Lió P, Crescenzi P, Fani R, Fondi M. 2015. MeDuSa: a multi-draft based scaffolder. Bioinformatics 31:2443–2451. doi:10.1093/bioinformatics/btv171. 25810435

[10] Mikheenko A, Prjibelski A, Saveliev V, Antipov D, Gurevich A. 2018. Versatile genome assembly evaluation with QUAST-LG. Bioinformatics 34:i142–i150. doi:10.1093/bioinformatics/bty266. 29949969 PMC6022658

[11] Jolley KA, Bliss CM, Bennett JS, Bratcher HB, Brehony C, Colles FM, Wimalarathna H, Harrison OB, Sheppard SK, Cody AJ, Maiden MCJ. 2012. Ribosomal multilocus sequence typing: universal characterization of bacteria from domain to strain. Microbiology (Reading) 158:1005–1015. doi:10.1099/mic.0.055459-0. 22282518 PMC3492749

[12] Tatusova T, DiCuccio M, Badretdin A, Chetvernin V, Nawrocki EP, Zaslavsky L, Lomsadze A, Pruitt KD, Borodovsky M, Ostell J. 2016. NCBI Prokaryotic Genome Annotation Pipeline. Nucleic Acids Res 44:6614–6624. doi:10.1093/nar/gkw569. 27342282 PMC5001611

[13] Blin K, Shaw S, Steinke K, Villebro R, Ziemert N, Lee SY, Medema MH, Weber T. 2019. antiSMASH 5.0: updates to the secondary metabolite genome mining pipeline. Nucleic Acids Res 47:W81–W87. doi:10.1093/nar/gkz310. 31032519 PMC6602434

